# Stereotactic Radiosurgery for Atrioventricular Node Ablation in Swine: A Study on Efficacy and Dosimetric Evaluation of Organs at Risk

**DOI:** 10.7759/cureus.18785

**Published:** 2021-10-14

**Authors:** Paul Ramia, Farah Ollaik, Lara Hilal, Wassim Jalbout, Wael AlJaroudi, Amin Al Ahmad, Pierre Sfeir, Abdo Jurjus, Marwan Refaat, Bassem Youssef

**Affiliations:** 1 Radiation Oncology, American University of Beirut, Beirut, LBN; 2 Cardiology, Clemenceau Medical Center, Beirut, LBN; 3 Cardiology, Texas Cardiac Arrhythmia Institute, St David’s Medical Center, Austin, USA; 4 Cardiothoracic Surgery, American University of Beirut, Beirut, LBN; 5 Anatomy, American University of Beirut, Beirut, LBN; 6 Cardiovascular Disease, American University of Beirut, Beirut, LBN

**Keywords:** stereotactic radiosurgery, swine model, av node ablation, ventricular tachycardia

## Abstract

Introduction

Stereotactic radiosurgery (SRS) delivered to arrhythmogenic foci within the heart is a promising treatment modality. We dosimetrically evaluated the radiation dose to the organs at risk of four swine that were successfully treated with linear-accelerator-based SRS for atrioventricular (AV) node ablation.

Materials and methods

Single‐chamber pacemakers were implanted in four large white breed swine. Cardiac computed tomography simulation scans were performed to localize the AV node and organs at risk. SRS (35-40 Gy) was delivered to the AV node, and the pigs were followed up with pacemaker interrogations. One-sample t-tests were used to evaluate Dmax of great vessels, esophagus, and chest wall as compared to known normal tissue constraints as per RTOG 0631 and AAPM Task Group 101.

Results

All pigs had disturbances of AV conduction with progressive transition into complete heart block. Macroscopic and microscopic evaluation showed fibrosis in the AV node but did not reveal any changes in non-nodal cardiac tissue or vessels. The mean Dmax±SD (p-value) of the chest wall (14.7±3.3 (0.02)), esophagus (10.7±1.1 (<0.01)) superior vena cava (3.3±4.1 (<0.01)), right pulmonary artery (16.1±6.4 (<0.01)), right pulmonary vein (15.7± 5 (<0.01)), left pulmonary artery (11.1±1.7 (<0.01)) and left pulmonary vein (14.1±2.6 (<0.01)), and the inferior vena cava (33.68±1.6 (0.026)) were significantly below the normal tissue constraint cutoffs. Mean±SD (p-value) of the ascending aorta (19.4±16.1 (0.12)) was not significantly different than normal tissue constraint cutoffs. One swine model treated at 40 Gy had small area of hotspot in the ascending aorta (40.65 (0.4 cc)).

Conclusion

We have demonstrated in our swine models that SRS using 35-40 Gy can be done without exceeding known human normal tissue constraints to the chest wall, esophagus, and great vessels.

## Introduction

Cardiac arrhythmias are a significant cause of morbidity and mortality with atrial fibrillation, affecting at least 2.3 million people in the United States alone [1]. Patients with cardiac arrhythmias often have multiple treatment options including medication as well as catheter ablation of arrhythmogenic foci [2]. Success rates for catheter ablation are relatively high with low risk of complications; however, several contraindications exist in performing this procedure in high-risk patients [3-5]. 

External beam radiation therapy has been previously utilized for treatment of benign condition. Specifically, it has been used in the setting of trigeminal neuralgia through ablation of the trigeminal nerve using high dose of radiation in a single fraction with high success rates [6]. In stereotactic radiosurgery (SRS), the delivery of high doses of radiation therapy in a single fraction to small targets with increased precision and image guidance can be achieved with either photon or particle therapy utilizing modern radiotherapy machines. SRS has now become an integral part of the treatment of numerous disease sites including lungs and intracranial tumors [7,8]. There have been multiple studies on animal models, trying to evaluate the feasibility of ablating arrhythmogenic foci in the heart, using this treatment modality and have showed that a dose of 25-40 Gy is sufficient to cause adequate scarring/ablation [9-11]. More recently, ENCORE-VT, a prospective phase I/II study using a single fraction of 25 Gy on 19 human patients with refractory ventricular tachycardia or cardiomyopathy related to premature ventricular contractions, showed reduction of arrhythmia burden and antiarrhythmic use [12].

Due to the novelty of this treatment modality, little is known about the dosimetric constraints to adjacent organs at risk, which are of importance for the procedure safety. In our study, we evaluated the radiation dose to the organs at risk of four pigs that were successfully treated with linear-accelerator-based SRS for atrioventricular (AV) node ablation.

This article was previously presented as a poster (https://www.redjournal.org/article/S0360-3016(18)32719-6/fulltext) on November 1, 2018.

## Materials and methods

**Figure 1:**
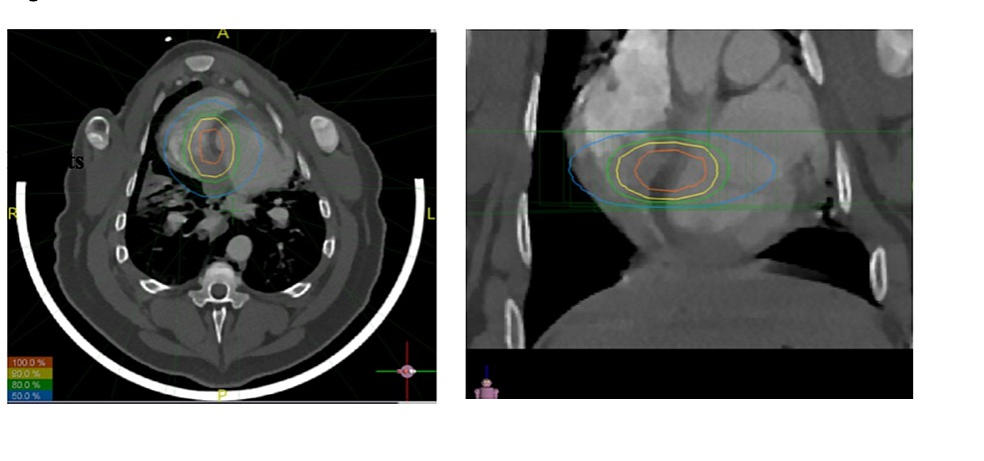
Left: Isodose lines in axial view; Right: isodose lines in coronal view

Single-chamber St Jude pacemakers were surgically inserted into four adult white breed swine (weight 40-75 kg; four females). The experiments were approved by Institutional Animal Care and Use Committee of the American University of Beirut. These pigs then underwent a cardiac computed tomography (CT) simulation with contrast under general anesthesia to localize the AV node. Two 4-dimensional CT sets were obtained in both diastolic and systolic phases with 1 mm slice reconstruction for treatment planning. Both the cardiologist and the radiation oncologist were present for contouring of the AV node on the different CT sets to generate an internal target volume, which was later expanded by 5 mm to establish a planning target volume. Forward planning using field-in-field technique to reduce inhomogeneity was done using Panther software (Prowess Inc, Concorde, CA) version 5.10. (Figure 1). Prior to treatment delivery, a cone-beam CT scan was obtained, and correctional shifts were done accordingly to establish millimeter accuracy. Treatment was delivered with nine coplanar beams on Siemens Artiste linear accelerator with 5-mm thickness multi-leaf collimators. Two of the pigs received a dose of 35 Gy, whereas the other two received 40 Gy. 

The organs at risk, namely the great vessels (ascending aorta, superior and inferior vena cava, right and left pulmonary arteries, right and left pulmonary veins), esophagus, and chest wall, were all contoured on the treatment planning software. Data of received doses were extracted from dose volume histograms and compared to known normal tissue constraints as per RTOG 0631 (Dmax great vessels <37 Gy; esophagus <16 Gy) and AAPM Task Group 101 (Dmax chest wall <22 Gy) [13,14].

The pigs were followed up with weekly pacemaker interrogations to observe for potential electrocardiographic changes. Once changes were observed, the pigs were euthanized, and cardiac tissues were sent for pathologic evaluation that has been previously reported [9].

One-sample t-tests were used and a two-sided p-value <0.05 was used uniformly to indicate significance. Statistical analysis was conducted using the SPSS version 23.0 software (SPSS Inc., Chicago, IL).

## Results

All four pigs had arrhythmogenic abnormalities, which transitioned into complete heart block within 12 weeks of SRS treatment with one of the pigs having a complete heart block within eight weeks. The timeframe and electrocardiographic (EKG) changes of AV node conduction have been previously described [9].

**Figure 2:**
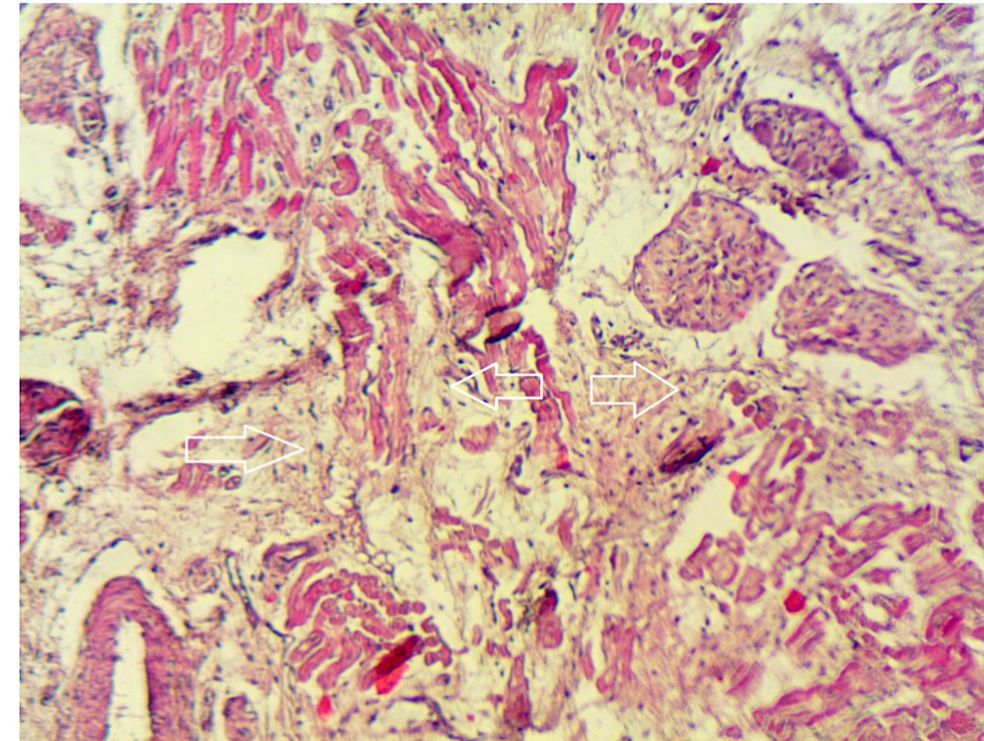
Pathology section showing fibrosis and loss of architecture in the atrioventricular node. The arrows point to areas of necrosis and loss of the specialized nodal cardiac muscle cells with fibrotic replacement (hematoxylin and eosin stained, magnification x250)

After euthanasia, representative tissue samples were taken and studied under light microscopy. Pathologic examination showed areas of fibrosis in the AV node of all four pigs with no evidence of fibrosis in any of the surrounding tissues (Figure 2).

None of the retrieved normal tissue dose-volume histogram parameters exceeded the established dose constraints except for the ascending aorta in one of the swine that was treated at 40 Gy. When the plan was reviewed, this was explained by the presence of a hotspot in the ascending aorta (40.65 (0.4 cc)). The maximal dose to the esophagus, chest wall, inferior and superior vena cava, and all the pulmonary vasculature were significantly less than the established dose constraints, whereas there were no significant differences between the maximal dose to the ascending aorta when compared to the control values. 

The mean Dmax ± SD (p-value) values were found to be chest wall (14.7±3.3 (0.02)), esophagus (10.7±1.1 (<0.01)), superior vena cava (3.3±4.1 (<0.01)), right pulmonary artery (16.1±6.4 (<0.01)) and right pulmonary vein (15.7±5 (<0.01)), and left pulmonary artery (11.1±1.7 (<0.01)) and left pulmonary vein (14.1±2.6 (<0.01)). The mean ± SD (p-value) values of ascending aorta and the inferior vena cava were found to be (19.4 ± 16.1 (0.12)) and (33.68 ± 1.6 (0.026)), respectively (Table 1).

**Table 1:** Maximal dose Dmax delivered to selected organs at risk (Gy) Abbreviations: AA, ascending aorta; Dmax, maximal dose; IVC, inferior vena cava; PA, pulmonary artery; PV, pulmonary vein; SVC, superior vena cava.

Pigs	AA	SVC	Right PA	Left PA	Right PV	Left PV	IVC	Esophagus
1	9.72	2.5	18.4	9.53	12.9	16.5	31.26	10.57
2	4.64	1.5	17.47	11.22	22.74	14.96	34.3	10.06
3	22.59	1.52	6.83	10.5	11.34	10.5	34.61	9.92
4	40.65	9.35	21.58	13.51	15.85	14.43	34.56	12.28

## Discussion

While catheter ablation has been shown to be a standard of care for cardiac arrhythmias with excellent outcomes [15,16], it may not be appropriate in some patients due to the location of arrhythmogenic focus or it might not be successful in some patients whose arrhythmias remain refractory to catheter ablation [5]. The procedural rates of complications from catheter ablation have decreased over time; however, it remains at roughly 3%, although this might differ by the target location as well as the institutional expertise. Complications can include thromboembolism, infection, hematoma, as well as new arrhythmias and even death [3,4]. Moreover, there are several documented cases of ablation failure that may be largely attributed to anatomic factors [17]. These factors provide justification to developing alternative, non-catheter-based methods of ablation in those patients who do not benefit from catheter ablation. 

This study further supports the growing body of evidence that SRS can be utilized for the ablation of cardiac arrhythmias in an effective manner. By studying the dosimetric properties of the swine that were treated with SRS and comparing them to historical normal tissue constraints, we were able to show that this type of procedure can be performed with acceptable radiation doses to the great vessels as well as the chest wall and the esophagus. 

Several strengths can be inferred from our study. We have shown that while targeting the AV node is radiologically challenging, it is feasible and reproducible as demonstrated by electrophysiological and histologic correlation. The histological correlation is unique because of the difficulty to obtain histological samples from collateral organs in patients who undergo stereotactic radioablation. Also, similar to the study by Cuculich et al. we have shown that using four-dimensional CT imaging as well as pretreatment cone-beam CT scans is adequate to successfully target the small areas within the myocardium using a linear accelerator. This is in contrast with Sharma et al. where robotic radiosurgery was performed using the CyberHeart system, which may not be readily available in many radiotherapy treatment centers worldwide [11,18].

Our study has several limitations. It was conducted in a single institution with data from treatment plans retrospectively evaluated. Another limitation is that we used human dose constraints for statistical comparison of data retrieved from swine treatment, since there are no known tissue constraints for swine models. Another weakness of our study is the small size and the lack of long-term clinical follow-up of the pigs. The pigs were sacrificed when AV block was achieved on electrocardiographs. 

Despite the numerous limitations of our study, it contributes to the growing body of evidence that stereotactic radiation therapy is feasible and effective in treating arrhythmogenic foci in the heart. This innovative treatment modality shows promise, especially for patients with ventricular tachycardias and who are not candidates for standard-of-care catheter ablative techniques or if they are refractory to catheter ablation. Recently, human data are being made available, with Cuculich et al. showing that in a small sample of patients with refractory ventricular tachycardia, SRS was able to achieve a reduction from baseline number of arrhythmias without any major short-term clinical complications [18,19]. Longer-term results from a phase I/II study of electrophysiology-guided noninvasive cardiac radioablation for treatment of ventricular tachycardia (ENCORE-VT) showed persistent success of radioablation in 78% of patients after more than two years of follow-up [12]. However, stereotactic radioablation is not curative and recurrent ventricular tachycardia might arise at the radiation treatment borderzone. Our study adds to the literature on the safety of stereotactic radioablation. Of note, one of the pigs received a higher-than-expected maximum dose to the ascending aorta (Dmax=40.56 Gy) that did not correlate to any histological or electrophysiological changes. By comparison, the ENCORE-VT trial employed a lower single-fraction dose of 25 Gy and reported doses received by the great vessels are expectedly low [20]. The excellent results of the patients enrolled in ENCORE-VT may justify the use of lower radiation dose than the one used in our study [12]. Safety profiles of stereotactic ablation in ventricular tachycardia are currently being studied in the ongoing RAVENTA trial [21].

## Conclusions

In conclusion, this study on swine animal models shows the efficacy and histologic correlation of SRS for the ablation of the AV node. This was done without exceeding known normal tissue constraints in humans and, therefore, adds to the emerging literature on cardiac SRS. More clinical data are required to better determine the long-term safety and efficacy of this promising treatment modality.
